# Comprehensive Evaluation of Fructus Tsaoko, Seeds of Tsaoko Fructus and Ginger Made Seeds of Tsaoko Fructus Based on Drug‐Like Compounds and Aroma Analysis

**DOI:** 10.1002/fsn3.71834

**Published:** 2026-05-02

**Authors:** Han Chen, Meiquan Yang, Weize Yang, Mingju Qi, Zongliang Xu, Tianmei Yang, Jinyu Zhang

**Affiliations:** ^1^ Medicinal Plants Research Institute Yunnan Academy of Agricultural Sciences Kunming China; ^2^ College of Forestry Southwest Forestry University Kunming China

**Keywords:** drug‐like and aroma metabolites, fructus tsaoko, ginger made seeds of Tsaoko Fructus, seeds of Tsaoko Fructus

## Abstract

Fructus tsaoko (CG) is the dried fruit of *Amomum tsaoko* Crevost et Lemaire. seeds of Tsaoko Fructus (QC) and ginger made seeds of Tsaoko Fructus (JZ) are two important decoction pieces derived from CG, which are rich in various chemical constituents. In this study, untargeted metabolomics combined with principal component analysis (PCA) was employed to establish models for analyzing and evaluating the drug‐likeness of CG, QC, and JZ. Meanwhile, the aroma characteristics and volatile components of these samples were analyzed using relative odor activity values (ROAVs) and quantitative descriptive analysis (QDA). GC–MS and LC–MS analyses identified 1625 and 1145 metabolites, respectively. Further screening revealed 108 metabolites with drug‐like properties and 44 aroma‐related compounds. The dominant drug‐like components were flavonoids and heterocyclic compounds. Paozhi (traditional Chinese herbal processing) mainly affected the relative contents of flavonoids and lignans. The drug‐likeness decreased in the order of CG < QC < JZ. Among the aroma components, ketones and heterocyclic compounds contributed most to the overall aroma. CG was characterized by a pungent aroma with a hint of green notes, and its key aroma components were 3‐octen‐2‐one, cyclohexanone, 2,2,6‐trimethylcyclohexanone, and (5Z)‐octa‐1,5‐dien‐3‐one. QC was characterized by a roasted aroma accompanied by fatty, green, and pungent notes, with 2‐methoxy‐3,5‐dimethylpyrazine, (5Z)‐octa‐1,5‐dien‐3‐one, and 3‐octen‐2‐one as the key aroma components. JZ was characterized by a pungent aroma with a hint of green notes, and its key aroma components were 1‐nonen‐3‐one, cyclohexanone, 2,2,6‐trimethylcyclohexanone, and 2‐methoxy‐3‐(2‐methylpropyl) pyrazine. This study provides new insights into the metabolic changes of CG, QC, and JZ, laying a theoretical foundation for their more refined utilization and high‐quality processing.

## Graphical abstract

This study mainly consists of two parts. First, after screening for drug‐like metabolites via LC–MS and GC–MS, a comprehensive evaluation model was established using a principal component analysis‐based weighted scoring method, by which the drug‐likeness ranking of three samples was determined. Second, key aroma compounds were screened from the GC–MS profiling by the relative odor activity value (ROAV) method, and combined with quantitative descriptive analysis (QDA), the key contributing aroma substances and flavor profiles of the three samples were comprehensively identified.

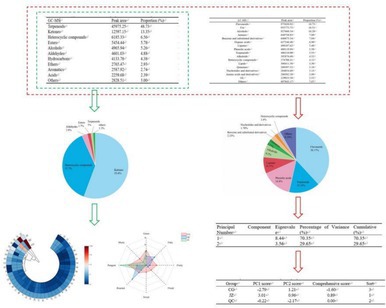

## 1 Introduction

Fructus tsaoko (CG) is the dried fruit of *Amomum tsaoko* Crevost et Lemaire, which has rich medicinal and edible value (Wen et al. 2025). Food serves to nourish the body, while medicine serves to eliminate disease. As a culinary ingredient, CG is employed to remove fishy and muttony off‐flavors from dishes, thereby enhancing their overall flavor profile (Wang 1981). As a traditional Chinese medicinal herb, it possesses a warm and pungent nature, acts upon the spleen and stomach channels, and belongs to the class of aromatic herbs that transform dampness (Tian et al. 2000). It is characterized by a strong aromatic odor and warm‐dry properties. It is traditionally used to break stagnation and relieve food retention, dispel cold and dampness, and treat a variety of symptoms including irritability, malaria, spleen and stomach deficiency‐induced diarrhea, abdominal distension and pain, vomiting, phlegm and food accumulation, and chills with fever (Wen et al. 2025). According to traditional Chinese medicine, it is believed that paozhi of CG pieces can mitigate its drying nature and enhance its warming effect on the middle burner, as well as its ability to dispel cold (Ni et al. 2023; Yu et al. 2023). *A. tsaoko* is characterized by a pungent aroma. Its fresh fruits are small and red, emitting a faint scent. In contrast, the mature dried fruits emit a strong, aromatic, and pungent odor (Imran et al. 2024). QC is a traditional Chinese medicine (TCM) decoction piece made from CG; JZ is made of CG as the main ingredient, ginger juice as the auxiliary material concoction of traditional Chinese medicine (China Pharmacopoeia Commission 2025).

CG contains a variety of chemical components, with volatile oil as the primary constituent. This oil is not only the source of its unique aroma but also its main active component, possessing various biological activities such as anti‐diabetic, gastroprotective, antioxidant, hypoglycemic, anti‐inflammatory, antibacterial, and anti‐tumor effects (He et al. 2024; He, Yang, and Wang 2023; He, Lin, et al. 2023). Regarding its applications in food, the biological activities of volatile oil play an important role in storage and preservation (Shang et al. 2022). For instance, it can serve as a potential natural food preservative, potentially extending the shelf life of food products in the future. Among the non‐volatile components, flavonoids and polyphenols are the predominant constituents (Shi et al. 2022). Flavonoids exhibit biological activities such as antioxidant, neuroprotective, anti‐diabetic, and anti‐inflammatory effects (Wang et al. 2021; Zhou et al. 2024). In particular, polyphenols demonstrate activities including anti‐obesity, regulation of high cholesterol, anti‐complementary activity, and antioxidant properties (Sun and Shahrajabian 2023; Wang et al. 2021). Although CG has a long history of clinical application, its value as a medicinal material has received considerably less attention than its use as a condiment. Therefore, it is particularly crucial to clarify the bioactive components of CG and its various herbal slices, and to establish an objective and comprehensive evaluation method. This is of great significance for both clinical application and paozhi technology.

Under heating conditions and auxiliary agents, the bioactivity and odor characteristics of Chinese medicinal slices prepared by paozhi change as the chemical composition varies (Changjiang 2015; Wu et al. 2018; Ren et al. 2024). Modern research shows that compared with CG, the sulfide response value of QC is significantly reduced, the total volatile oil content is decreased, the potential biological toxicity and carcinogenic components such as trans‐2‐heptanoate ethyl and trans‐2‐decene disappear, and new active components such as 1S‐α‐pinene, 4⁃terpene alcohol, ociene and junene are added (Ren et al. 2024). The non‐volatile components of CG are easier to seep out after paozhi are more likely to seep out, and the content is mainly flavonoids and polyphenols (Hu et al. 2024; Lu et al. 2018). The results showed that there were obvious differences in the odor characteristics of different TCM decoction pieces of CG, and the odor difference was mainly reflected in the three sensors of W5S, W1W, and W2W, and the odor and volatile components of different TCM decoction pieces were different, which may be related to the change of the proportion of volatile components in the paozhi process of CG (Ren et al. 2024; Chi et al. 2021). Therefore, it is great significance to analyze the differences in medicinal properties and aroma of CG and its different TCM decoction pieces for the development and utilization of its medicinal and edible resources.

Metabolomics is an important tool for the analysis of compounds in samples, and gas chromatography–mass spectrometry (GC–MS) and liquid chromatography–mass spectrometry (LC–MS) are commonly used for the separation and identification of complex components, which have the advantages of high sensitivity and fast analysis speed (Wu, Zhang, Zheng, et al. 2022). LC–MS has been widely used in variety identification and pesticide residue detection (Chen et al. 2023). As technology advances, more research is being done to combine GC–MS and LC–MS with other analytical methods. The contribution of aroma compounds to the flavor is different, and the contribution rate of aroma components to the overall aroma of the sample cannot be accurately judged based on the content of the aroma components (Wang et al. 2022). The relative odor activity value (ROAV) is a method established by combining the sensory thresholds of compounds to identify key flavor compounds in food, and is used to elucidate the contribution of each aroma compound to the overall aroma profile of a sample. The quantitative descriptive analysis (QDA) is a sensory evaluation method widely employed to quantify the aroma profiles of products, particularly spices. Through the establishment of a scientific lexicon database and a standardized evaluation system, it translates subjective olfactory perceptions into quantifiable data.

In order to better analyze the drug‐like and aroma components in CG, QC, and JZ, this paper clarified the differences of drug‐like components based on GC–MS and LC–MS, and on this basis, the objective and comprehensive evaluation of their drug‐like properties was carried out; based on GC–MS components, combined with ROAV and QDA methods, the aroma characteristics of CG, QC, and JZ were analyzed. The purpose of this study was to lay a theoretical foundation for the difference of efficacy and the mechanism of aroma formation in the process of understanding the medicinal components and aroma substances of CG, QC, and JZ, and the research results could provide a reference for the production of high‐quality CG, QC, and JZ, the rational use of their efficacy and the improvement of flavor quality.

## 2 Materials and Methods

### 2.1 Plant Materials

Approximately 10 kg of uniformly ripe fruits were harvested from plantations in Dehong, Yunnan Province. All fruits were identified by Dr. Jinyu Zhang (Medicinal Plants Research Institute, Yunnan Academy of Agricultural Sciences, Kunming, China) as the fruits of *A. tsaoko* in Zingiberaceae.

The collected *A. tsaoko* was washed, dried in an electric constant‐temperature blast oven at 70°C for 24 h, then removed and randomly divided into three samples for subsequent processing. The first sample, after impurity removal, yielded sample CG. For the second sample, impurities were removed, the fruit shells were stripped off, and then the sample was stir‐fried in a drum‐type herbal stir‐fry at 160°C for 15 min, resulting in sample QC. The third sample underwent impurity and fruit shell removal, followed by the addition of ginger juice (1/10 w/w of the sample) for a 30‐min moistening period; subsequently, it was stir‐fried in a drum‐type herbal stir‐fry at 140°C for 3 min to obtain sample JZ (China Pharmacopoeia Commission 2025; Chen et al. 2025).

### 2.2 GC–MS Conditions

Analysis was performed by HS‐SPME‐GC‐MS under the following chromatographic conditions: DB‐5MS capillary column (30 m × 0.25 mm × 0.25 μm, Agilent J&W Scientific, Folsom, CA, USA) with high‐purity helium as carrier gas at 1.2 mL/min, injection port temperature at 250°C, ramp program: 40°C for 3.5 min, 10°C/min to 100°C, and 7°C/min rise to 180°C, and finally 25°C/min to 280°C for 5 min. Mass spectrometry conditions: electron bombardment ion source (EI), ion source temperature 230°C, quadrupole temperature 150°C, mass spectrometry interface temperature 280°C, electron energy 70 eV, scanning mode selective ion detection mode (SIM), qualitative and quantitative ion precision scanning.

### 2.3 LC–MS Conditions

Analysis was performed using UCPLC–MS/MS. Liquid phase conditions: Chromatographic Column: Waters ACQUITY UPLC HSS T3 Column 1.8 μm 2.1 mm × 100 mm; Mobile phase: Phase A is ultrapure water (with 0.1% formic acid added), and Phase B is acetonitrile (with 0.1% formic acid added). Column temperature: 40°C Flow rate: 0.40 mL/min injection volume: 4 μL. The mass spectrometry conditions mainly include: The temperature of the electrospray ionization (ESI) source is 550°C, the mass spectrometry voltage is 5000 V (positive mode), −4000 V (negative mode), the ion source gas I (GS I) is 50 psi, and the gas II (GS II) is 60 psi. The Curtain Gas (psi) is 35 psi, and the collision‐activated dissociation (CAD) parameter is set to high during collision. In the triple quadrupole (Qtrap), each particle pair is scanned and detected based on the optimized declutering potential (DP) and collision energy (CE).

### 2.4 Compound Identification and Quantification

#### 2.4.1 GC–MS Metabolites

Metabolite data, including retention times and peak areas, were retrieved from published literature and a self‐constructed database. Isotopic internal standards were employed, and the relative abundances of volatile metabolites in the samples were calculated using the following Equation (1):

(1) $$X_{i}=\frac{V_{s}\times C_{s}}{M}\times \frac{I_{i}}{I_{s}}\times 10^{-3}$$

where *X*_*i*_ is the content of compound *i* in the sample (μg/g); *V*_*s*_ is the volume of added internal standard (μL); *C*_*s*_ is the concentration of the internal standard (μg/mL); *M* is the mass of the sample (*g*); *I*_*s*_ is the peak area of the internal standard; and *I*_*i*_ is the peak area of compound *i* in the sample.

#### 2.4.2 LC–MS Metabolites

The raw mass spectrometry data were converted to mzML format using ProteoWizard and processed with XCMS for peak detection, alignment, retention time correction, and normalization. Peaks with a missing value rate > 50% across sample groups were removed, and the remaining peak areas were corrected using the SVR method. Metabolites were identified from the corrected peaks by searching a self‐constructed laboratory database, integrating public and in silico databases, and applying the metDNA approach (Level: annotation confidence (2: indicating 70% confidence; 3: indicating 50% confidence); score: annotation confidence score). Subsequently, data from positive and negative ionization modes were merged, retaining the feature with the highest annotation level and lowest CV. Finally, features with an annotation score > 0.50 (0.50–0.60: low confidence; 0.60–0.80: medium confidence; 0.80–1.00: high confidence) and QC CV < 0.5 were retained, yielding relative metabolite abundances suitable for inter‐sample comparisons. Detailed information regarding the LC–MS components is provided in Table S2.

#### 2.4.3 Durg‐Like Metabolites

The chemical constituents identified by GC–MS and LC–MS were matched to their corresponding entries in the Traditional Chinese Medicine Systems Pharmacology Database (TCMSP, http://tcmspw.com/tcmsp.php) using their Chemical Abstracts Service (CAS) numbers. For each compound, we retrieved its TCMSP ID, oral bioavailability (OB), and drug‐likeness (DL) scores. Compounds meeting the thresholds of OB ≥ 20% and DL ≥ 0.15 were then selected as drug‐like candidates (Guo et al. 2025; Ru et al. 2014).

### 2.5 Construction of Evaluation Model for Durg‐Like

A comprehensive evaluation model was constructed using a PCA‐based weighted scoring method. After standardizing the indicators, principal component analysis (PCA) was performed to extract the principal components. The variance contribution rate of each component served as the objective weight, and the comprehensive score was calculated by a weighted sum (Wu, Zhang, Yan, et al. 2022).

The calculation process is as follows (Wei et al. 2022):

Determination of the cumulative variance contribution rate of PC1 and PC2: The cumulative variance contribution rates of PC1 and PC2 ($\mathrm{PC}1_{i}$ and $\mathrm{PC}2_{i}$) were calculated from the variance contribution rates of the two principal components (PC1 and PC2) in the original PCA indicator data, and the calculation formula (2) is as follows:

(2) $$\mathrm{PC}1_{i}=\frac{\mathrm{PC}1}{\mathrm{PC}1+\mathrm{PC}2}；\mathrm{PC}2_{i}=\frac{\mathrm{PC}2}{\mathrm{PC}1+\mathrm{PC}2}$$

Calculation of the comprehensive score: Based on $\mathrm{PC}1_{i}$, $\mathrm{PC}2_{i}$, and the scores of PC1 and PC2 (PC1score and PC2score) of each sample in the original PCA indicator data, the comprehensive score *F* of each sample was calculated, and the calculation formula (3) is as follows:

(3) $$F_{i}=\mathrm{PC}1_{i}\times \mathrm{PC}1{\text{score}}_{i}+\mathrm{PC}2_{i}\times \mathrm{PC}2{\text{score}}_{i}$$

where i is the sample CG, QC, and JZ.

### 2.6 ROAV

The ROAV method was used to quantify the contribution of different GC–MS components to the overall flavor of the samples. The larger the ROAV value, the greater the contribution of the component to the overall flavor of the samples, and the ROAV values of all volatile compounds do not exceed 100. According to the degree of aroma contribution, ROAV can be divided into three ranges: 0.10 ≤ ROAV < 1, 1 ≤ ROAV < 100, and ROAV = 100. The ROAV value of each GC–MS component was calculated by formula (4) based on the relative quantification of each GC–MS component and the aroma threshold of each GC–MS component in water from references and databases (Wang et al. 2022).

(4) $$\text{ROAV}\approx \frac{C_{i}}{C_{\max}}\times \frac{T_{\max}}{T_{i}}\times 100$$

where $C_{i}$ is the relative content of each GC–MS component (%); $T_{i}$ is the sensory threshold of each GC–MS component (μg/kg); $C_{\max}$ is the relative content of the GC–MS component that contributes the most to the aroma of CG; $T_{\max}$ is the sensory threshold corresponding to the GC–MS component that contributes the most to the aroma of CG.

### 2.7 QDA

A quantitative descriptive sensory analysis was established based on the volatile components identified by GC–MS, aroma characteristics, and ROAV values. 9 evaluators with normal sensory sensitivity, who passed the screening and training, were selected to form a sensory evaluation team. Reasonable aroma attributes were determined as evaluation indicators by combining the sample aroma characteristics and relevant literature (Li et al. 2024). The aroma characteristics are shown in Table S1. A 10‐point scale was adopted for scoring: 0 indicated no odor detected, 1–3 indicated a weak odor, 4–6 indicated a moderate odor, and 7–10 indicated a strong odor. The evaluators performed olfactory testing in an odor‐free, constant temperature and humidity environment and conducted quantitative scoring. Each sample was measured in triplicate, and the average value was taken.

### 2.8 Statistical Analysis

Microsoft Excel 2019 was used for data organization and table generation. One‐way analysis of variance (ANOVA) on the relative contents of chemical components was performed using SPSS 27.0. Origin 2024 was utilized for graphical representation and PCA analysis. Heatmaps were generated with the pheatmap package in R (www.r‐project.org). Metabolite partial least squares discriminant analysis (PLS‐DA) and cross‐validation analysis of variance (CV‐ANOVA) were conducted using SIMCA 14.1.

## 3 Results and Discussion

### 3.1 Metabolites Analysis of LC–MS

#### 3.1.1 Sample Quality Control (QC) Analysis

**FIGURE 1:**
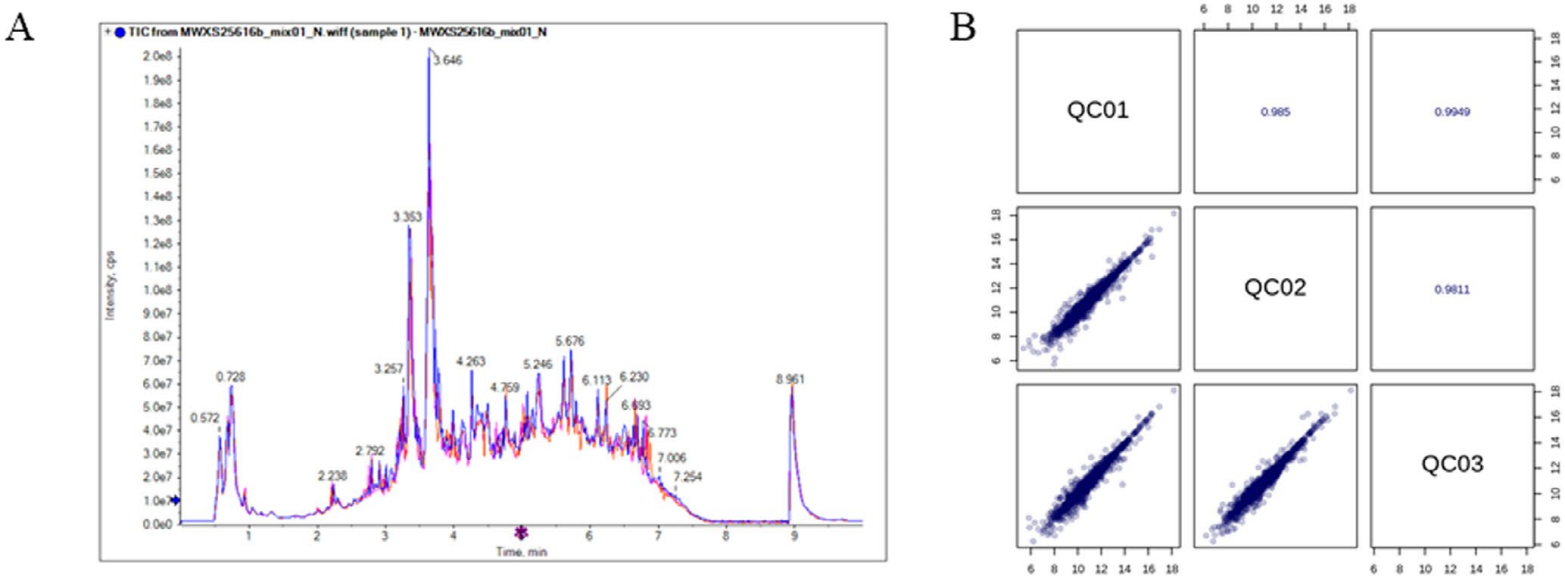
Sample quality control analysis. (A) Total ion current (TIC) chromatogram. (B) Pearson correlation analysis.

A QC sample was inserted after every 10 test samples to monitor analytical repeatability. The total ion chromatograms (TICs) of different QC samples, as shown in Figure 1A, exhibit high overlap in both retention time and peak intensity, indicating stable mass spectrometric signals for repeated measurements of the same sample. Pearson correlation analysis of the QC samples (Figure 1B) yielded |*r*| > 0.9800 (the closer |*r*| is to 1, the stronger the correlation), confirming the stability of the entire detection process and the high quality of the data.

#### 3.1.2 LC–MS Metabolites Identification

Non‐volatile components in CG, QC, and JZ were detected using ultra‐performance liquid chromatography–tandem mass spectrometry (UPLC–MS/MS). By comparing the mass spectra with metabolite databases, a total of 1145 metabolites were identified. The relative content percentages of various compound classes are presented in Table 1. Among them, flavonoids were the most abundant, accounting for 10.73%, followed by fatty acids (FA) (10.51%), alcohols (10.20%), amines (7.09%), benzene and substituted derivatives (7.04%), organic acids (6.90%), lignans (5.48%), phenolic acids (5.38%), terpenoids (5.35%), alkaloids (4.32%), heterocyclic compounds (4.12%), lipids (3.30%), quinones (3.18%), nucleotides and derivatives (3.13%), amino acids and derivatives (3.09%), glycosides (GL) (2.52%), with the remaining compounds present at relatively lower levels. These findings indicate that flavonoid compounds were the dominant class of volatile metabolites in CG, QC, and JZ, which aligns with previous research (Hu, Gao, Su, et al. 2023).

**TABLE 1:** The type, relative content and proportion of LC–MS metabolites.

LC–MS	Peak area	Proportion (%)
Flavonoids	975,630.92	10.73
FA	955,773.75	10.51
Alcohols	927,668.54	10.20
Amines	644,738.83	7.09
Benzene and substituted derivatives	640,675.34	7.04
Organic acids	627,246.48	6.90
Lignans	498,107.62	5.48
Phenolic acids	489,119.94	5.38
Terpenoids	486,216.00	5.35
Alkaloids	392,876.68	4.32
Heterocyclic compounds	374,708.41	4.12
Lipids	300,411.50	3.30
Quinones	289,397.32	3.18
Nucleotides and derivatives	284,834.69	3.13
Amino acids and derivatives	280,582.58	3.09
GL	229,033.56	2.52
Others	697,843.17	7.67

#### 3.1.3 PCA Analysis of LC‐MS Metabolites

**FIGURE 2:**
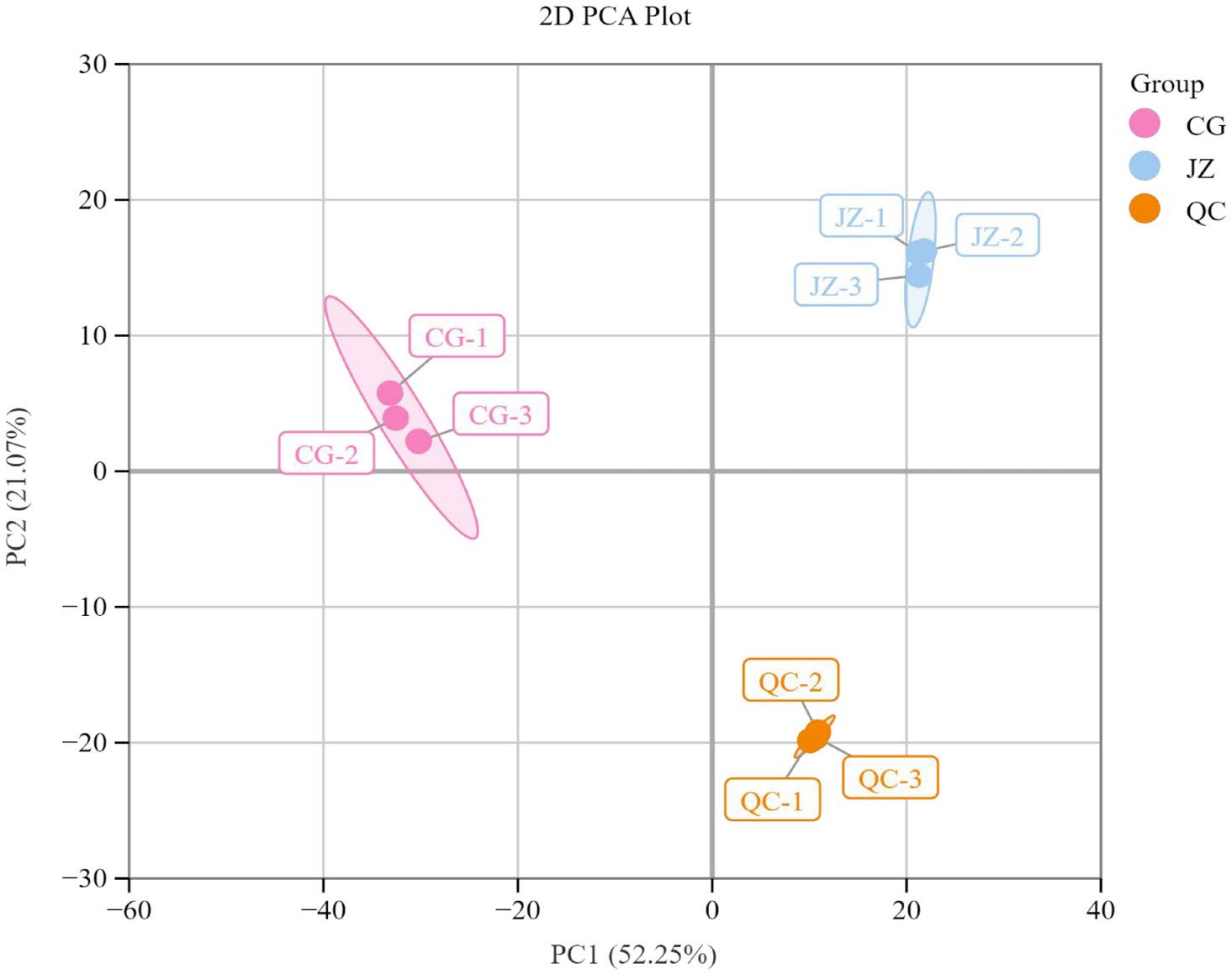
PCA plot of mass spectrometry data of CG, QC, and JZ.

Principal Component Analysis (PCA) is an unsupervised multivariate statistical method for pattern recognition, which helps elucidate overall metabolite differences between groups and the degree of variation within groups, revealing the internal structure among multiple variables through a few principal components. As shown in the PCA score plot (Figure 2), the distances between CG, QC, and JZ are quite large, indicating significant sample variability and clear metabolite classification trends. Samples within each group are evenly distributed and closely clustered, suggesting minimal intra‐group differences. The first principal component (PC1) and the second principal component (PC2) explain 55.25% and 21.07% of the total variability, respectively, with a cumulative contribution rate of 76.32%.

### 3.2 Metabolites Analysis of GC–MS

Volatile metabolites in CG, QC, and JZ were analyzed using headspace solid‐phase microextraction coupled with gas chromatography–mass spectrometry (HS‐SPME‐GC‐MS). By comparing the mass spectra with metabolite databases, a total of 1625 metabolites were identified. The relative content percentages of various compound classes are presented in Table 2. Among them, terpenoids were the most abundant, accounting for 48.73%, followed by ketones (13.35%), heterocyclic compounds (6.56%), esters (5.78%), alcohols (5.26%), aldehydes (4.88%), hydrocarbons (4.38%), ethers (2.93%), aromatics (2.74%), and acids (2.39%), with the remaining compounds present at relatively lower levels. These findings indicate that terpenoid compounds are the dominant volatile metabolites in CG, QC, and JZ, which aligns with previous research (Ren et al. 2024; Chi et al. 2021).

**TABLE 2:** The type, relative content and proportion of GC–MS metabolites.

GC–MS	Peak area	Proportion (%)
Terpenoids	45,975.25	48.73
Ketones	12,597.15	13.35
Heterocyclic compounds	6185.33	6.56
Esters	5454.44	5.78
Alcohols	4965.94	5.26
Aldehydes	4601.03	4.88
Hydrocarbons	4133.78	4.38
Ethers	2765.47	2.93
Aromatics	2587.92	2.74
Acids	2259.68	2.39
Others	2828.51	3.00

#### 3.2.1 PCA Analysis of GC–MS Metabolites

**FIGURE 3:**
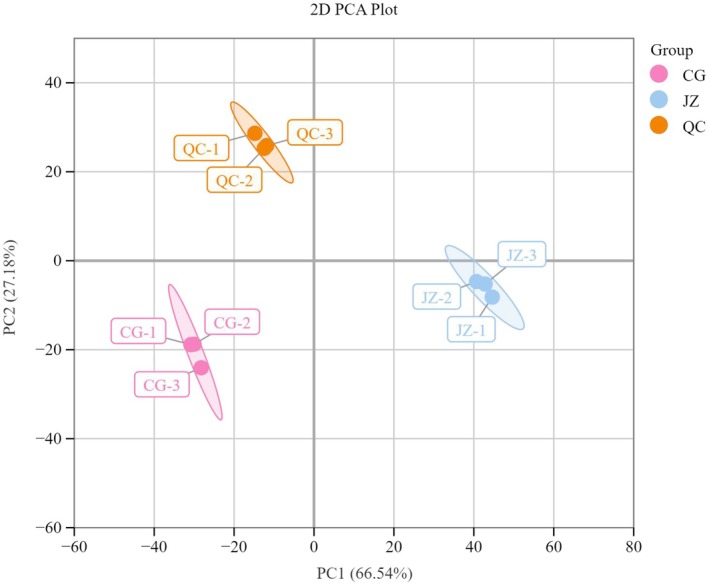
PCA plot of mass spectrometry data of CG, QC, and JZ.

The PCA score plot (Figure 3) illustrates distinct separations among CG, QC, and JZ, reflecting significant differences between the sample groups and clear trends in metabolite classification. Within each group, the samples are uniformly distributed and closely clustered, indicating minimal intra‐group variability. The first principal component (PC1) and the second principal component (PC2) account for 66.54% and 27.18% of the total variance, respectively, with a cumulative contribution rate of 93.72%.

### 3.3 Analysis of Durg‐Like Metabolites

Based on GC–MS and LC–MS data, and using OB ≥ 20% and DL ≥ 0.15 as screening thresholds, we identified 108 potential drug‐like metabolites (Table 3). These were categorized into Alkaloids (15 metabolites), Benzene and substituted derivatives (5 metabolites), Coumarins (5 metabolites), Flavonoids (23 metabolites), Heterocyclic compounds (3 metabolites), Lignans (9 metabolites), Lipids (4 metabolites), Nucleotides and derivatives (3 metabolites), Phenolic acids (9 metabolites), Quinones (3 metabolites), Terpenoids (14 metabolites), and Others (15 metabolites). As illustrated in Figure 1, Flavonoids dominate with a relative content of 38.17%, followed by Terpenoids (15.34%) and Phenolic acids (14.8%). The other classes, including Alkaloids (6.3%) and Lignans (8.17%), exhibited moderate levels. Collectively, these findings highlight Flavonoids as the primary active constituents across CG, JZ, and QC.

**TABLE 3:** 108 Kinds of medicinal ingredients.

Number	Compounds	Class	CAS	Molecule ID	OB%	DL
1	Cyclopamine	Alkaloids	4449‐51‐8	MOL009027	55.42	0.82
2	Napelline	Alkaloids	5008‐52‐6	MOL004759	34.48	0.71
3	Karakoline	Alkaloids	39‐089‐30‐0	MOL002397	51.73	0.73
4	Hypaconitine	Alkaloids	6900‐87‐4	MOL000538	31.38	0.26
5	Tetrahydroalstonine	Alkaloids	83‐45‐4	MOL009619	25.32	0.75
6	(‐)‐Berbine	Alkaloids	131‐10‐2	MOL008182	54.69	0.26
7	Quinine	Alkaloids	130‐95‐0	MOL009137	55.88	0.40
8	Bulbocapnine	Alkaloids	298‐45‐3	MOL000781	33.45	0.69
9	Rotundine	Alkaloids	10097‐84‐4	MOL006420	22.03	0.70
10	Songorine	Alkaloids	509‐24‐0	MOL002431	24.46	0.72
11	Nuciferine	Alkaloids	475‐83‐2	MOL007213	34.43	0.40
12	Corynantheine	Alkaloids	18904‐54‐6	MOL008478	31.94	0.64
13	Bicuculline	Alkaloids	485‐49‐4	MOL000791	69.67	0.88
14	Indirubin	Alkaloids	479‐41‐4	MOL002309	48.59	0.26
15	Ellipticine	Alkaloids	519‐23‐3	MOL009135	30.82	0.29
16	2‐[6‐(2‐Hydroxy‐2‐phenylethyl)‐1‐methyl‐2‐piperidyl]‐1‐phenylethanol	Benzene and substituted derivatives	552‐72‐7	MOL012207	60.53	0.32
17	Leiocarposide	Benzene and substituted derivatives	71953‐77‐0	MOL012172	28.15	0.65
18	2‐Cyclohexyl‐2‐hydroxy‐2‐phenylacetic acid	Benzene and substituted derivatives	4335‐77‐7	MOL009674	25.16	0.57
19	Populin	Benzene and substituted derivatives	99‐17‐2	MOL001371	108.89	0.20
20	Isobutyrylmallotochromene	Benzene and substituted derivatives	116964‐16‐0	MOL003849	22.80	0.69
21	Decursin	Coumarins	5928‐25‐6	MOL013077	39.27	0.38
22	Ostruthin	Coumarins	148‐83‐4	MOL003626	30.65	0.23
23	(2S)‐2‐(2‐hydroxypropan‐2‐yl)‐9‐[(2S,3R,4S,5S,6R)‐3,4,5‐trihydroxy‐6‐(hydroxymethyl)oxan‐2‐yl]oxy‐2,3‐dihydrofuro[3,2‐g]chromen‐7‐one	Coumarins	20320‐81‐4	MOL013101	70.10	0.20
24	Dalbergin	Coumarins	482‐83‐7	MOL002966	78.11	0.20
25	Marmesin	Coumarins	13849‐08‐6	MOL001944	50.28	0.17
26	Farrerol	Flavonoids	24211‐30‐1	MOL012432	42.65	0.26
27	L‐Epicatechin	Flavonoids	7295‐85‐4	MOL000492	54.83	0.24
28	(‐)‐Epiafzelechin	Flavonoids	24808‐04‐6	MOL006775	23.74	0.21
29	Cianidanol	Flavonoids	154‐23‐4	MOL000096	49.68	0.24
30	6″‐O‐Malonylglycitin	Flavonoids	137705‐39‐6	MOL011691	30.40	0.81
31	Icaritin	Flavonoids	118525‐40‐9	MOL004373	45.11	0.44
32	Didymin	Flavonoids	14259‐47‐3	MOL005849	38.55	0.24
33	Naringenin	Flavonoids	480‐41‐1	MOL004328	59.29	0.21
34	Licochalcone a	Flavonoids	58749‐22‐7	MOL000497	40.79	0.29
35	Glabranin	Flavonoids	41983‐91‐9	MOL004910	52.90	0.31
36	(‐)‐Epicatechin gallate	Flavonoids	1257‐08‐5	MOL006504	53.57	0.75
37	Tephrosin	Flavonoids	76‐80‐2	MOL013198	27.80	0.88
38	Isoginkgetin	Flavonoids	548‐19‐6	MOL002511	21.56	0.58
39	Pseudobaptigenin	Flavonoids	90‐29‐9	MOL000507	70.12	0.31
40	Rhamnazin	Flavonoids	552‐54‐5	MOL000351	47.14	0.34
41	Isorhamnetin	Flavonoids	480‐19‐3	MOL000354	49.60	0.31
42	Quercetin	Flavonoids	117‐39‐5	MOL000098	46.43	0.28
43	Taxifolin	Flavonoids	480‐18‐2	MOL004576	57.84	0.27
44	Epigallocatechin	Flavonoids	970‐74‐1	MOL006791	24.18	0.27
45	Plantagoside	Flavonoids	78708‐33‐5	MOL007813	58.12	0.28
46	Liquiritin	Flavonoids	551‐15‐5	MOL004903	65.69	0.74
47	Euxanthone	Flavonoids	529‐61‐3	MOL003358	92.98	0.16
48	3‐Hydroxyflavone	Flavonoids	577‐85‐5	MOL010246	47.91	0.16
49	Fastigilin B	Heterocyclic compounds	1861221	MOL012433	109.45	0.40
50	4‐Prenylresveratrol	Heterocyclic compounds	61517‐87‐1	MOL003879	40.54	0.21
51	Protoporphyrin IX	Heterocyclic compounds	553‐12‐8	MOL000783	30.86	0.56
52	2,3‐Bis(1,3‐benzodioxol‐5‐ylmethyl)butane‐1,4‐diol	Lignans	24563‐03‐9	MOL013192	49.64	0.46
53	Fargesone A	Lignans	116424‐69‐2	MOL012130	59.37	0.51
54	Yangambin	Lignans	13060‐14‐5	MOL007563	57.53	0.81
55	Honokiol	Lignans	35354‐74‐6	MOL005955	60.67	0.15
56	Diphyllin	Lignans	22055‐22‐7	MOL001699	36.23	0.75
57	Phyllanthin	Lignans	10351‐88‐9	MOL006812	33.31	0.42
58	Silandrin	Lignans	70815‐32‐6	MOL007455	64.14	0.94
59	Gomisin N	Lignans	82467‐52‐5	MOL003213	20.16	0.75
60	(+)‐Eudesmin	Lignans	526‐06‐7	MOL009047	33.29	0.62
61	Eicosapentaenoic acid	Lipids	10417‐94‐4	MOL010485	45.66	0.21
62	FFA(19:1)	Lipids	73033‐09‐7	MOL012894	29.84	0.17
63	Ganoderic acid H	Lipids	98665‐19‐1	MOL011196	24.81	0.73
64	FFA(18:2)	Lipids	60‐33‐3	MOL000432	45.01	0.15
65	2′‐Deoxyadenosine	Nucleotides and derivatives	958‐09‐8	MOL009000	30.13	0.15
66	Guanosine	Nucleotides and derivatives	118‐00‐3	MOL002687	21.43	0.21
67	Uridine‐5′‐diphosphate‐glucose	Nucleotides and derivatives	133‐89‐1	MOL013048	28.47	0.67
68	Gingerol	Phenolic acids	23513‐14‐6	MOL002467	35.64	0.16
69	Shogaol	Phenolic acids	36700‐45‐5	MOL006133	23.77	0.19
70	Ginkgolic acid	Phenolic acids	22910‐60‐7	MOL011051	20.18	0.32
71	Tachioside	Phenolic acids	109194‐60‐7	MOL007386	20.98	0.19
72	Cynarine	Phenolic acids	30964‐13‐7	MOL007326	31.76	0.68
73	Bis(2‐ethylhexyl) phthalate	Phenolic acids	117‐81‐7	MOL001490	43.59	0.35
74	Moracin M	Phenolic acids	56317‐21‐6	MOL004574	54.54	0.16
75	Agnuside	Phenolic acids	11027‐63‐7	MOL007264	22.01	0.83
76	Methylarbutin	Phenolic acids	6032‐32‐2	MOL000562	25.62	0.27
77	Danthron	Quinones	117‐10‐2	MOL006478	28.74	0.19
78	Aloin	Quinones	5133‐19‐7	MOL005060	22.18	0.71
79	Rhein	Quinones	478‐43‐3	MOL002268	47.07	0.28
80	Ailanthone	Terpenoids	981‐15‐7	MOL006284	27.96	0.74
81	Limonin	Terpenoids	1180‐71‐8	MOL003959	21.30	0.57
82	Swertiamarin	Terpenoids	17388‐39‐5	MOL003166	21.90	0.42
83	Corosolic acid	Terpenoids	4547‐24‐4	MOL010601	22.79	0.74
84	Triptophenolide	Terpenoids	74285‐86‐2	MOL003196	48.5	0.44
85	Ganoderic acid A	Terpenoids	81907‐62‐2	MOL011179	22.66	0.81
86	CID 6443057	Terpenoids	34420‐19‐4	MOL005230	50.76	0.34
87	Ingenol	Terpenoids	30220‐46‐3	MOL005225	27.34	0.43
88	Biosone	Terpenoids	471‐53‐4	MOL004804	22.05	0.74
89	(all‐E)‐Crocetin	Terpenoids	27876‐94‐4	MOL001406	35.30	0.26
90	Kirenol	Terpenoids	52659‐56‐0	MOL000144	23.18	0.39
91	Harpagoside	Terpenoids	19210‐12‐9	MOL009041	21.72	0.32
92	Caryophyllene oxide	Terpenoids	1139‐30‐6	MOL001193	45.75	0.15
93	Bicyclo[3.1.1]hept‐2‐en‐6‐one, 2,7,7‐trimethyl‐	Terpenoids	473‐06‐3	MOL008927	22.60	0.45
94	1‐O‐Acetylbritannilactone	Others	33627‐41‐7	MOL004089	30.12	0.22
95	Bis(2‐ethylhexyl) adipate	Others	103‐23‐1	MOL002030	29.15	0.27
96	Folic acid	Others	59‐30‐3	MOL000433	68.96	0.71
97	Rhapontigenin	Others	500‐65‐2	MOL002262	76.25	0.15
98	Dihydroquercetin	Others	98006‐93‐0	MOL004580	66.44	0.27
99	Rapanone	Others	573‐40‐0	MOL010973	34.15	0.24
100	N‐Acetylgalactosamine 4,6‐disulfate	Others	52510‐51‐7	MOL004569	23.04	0.24
101	Bowdichione	Others	53774‐75‐7	MOL002973	55.78	0.28
102	Capillarisin	Others	56365‐38‐9	MOL008043	57.56	0.31
103	Demethylwedelolactone	Others	6468‐55‐9	MOL003402	72.13	0.43
104	Cystathionine	Others	61135‐95‐3	MOL002989	48.41	0.43
105	Methyl ricinoleate	Others	141‐24‐2	MOL010205	36.30	0.19
106	3,7,11,15,23‐Pentaoxolanost‐8‐en‐26‐oic acid	Others	98665‐14‐6	MOL011190	27.13	0.81
107	Cymarin	Others	508‐77‐0	MOL001885	23.59	0.64
108	Procyanidin B1	Others	20315‐25‐7	MOL000004	67.87	0.66

**FIGURE 4:**
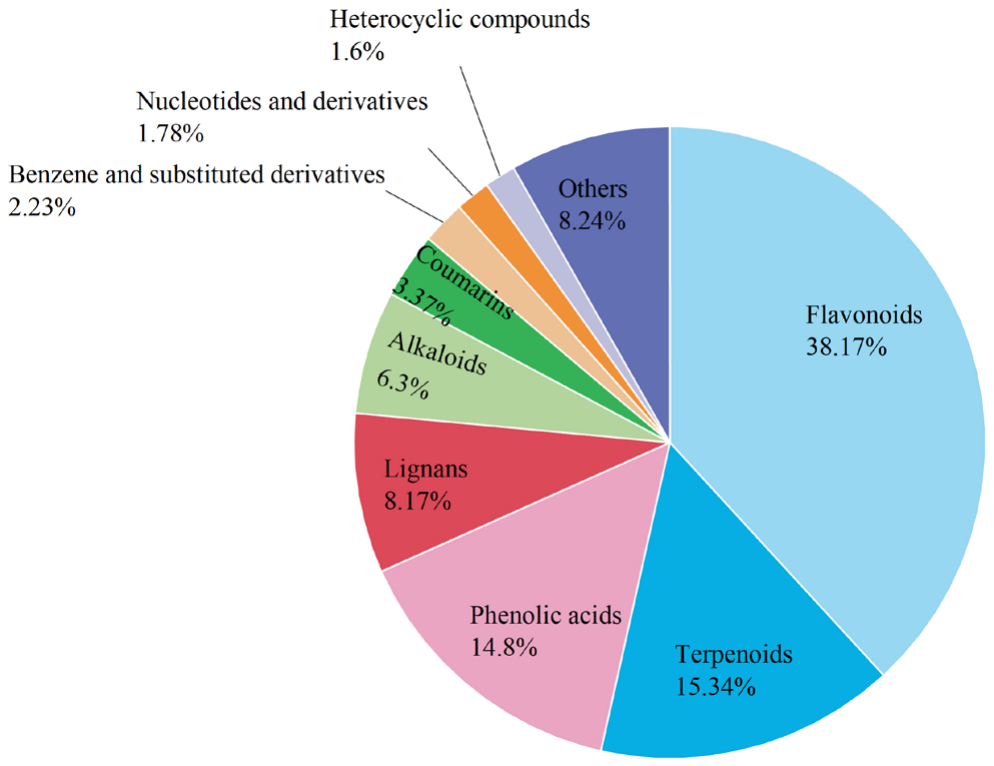
Relative content ratio of drug‐like components.

Figure 4 further reveals that flavonoid, phenolic acid, and terpenoid compounds maintain the highest relative contents across the three samples, while alkaloid and lignan compounds follow. Notably, the relative abundance of coumarin, flavonoid, phenolic acid, and terpenoid compounds increases sequentially from JZ to QC to CG. Conversely, alkaloid and benzene derivative compounds peak in CG, and lignan compounds reach their maximum in QC. The remaining metabolites display relatively low and stable concentrations.

Coumarin, flavonoid, phenolic acid, and Terpenoid compounds are highly susceptible to oxidation and decomposition under high‐temperature conditions. In the oxidative environment of the maillard reaction, they can be transformed into brown pigments or volatile degradation products, resulting in their relatively low concentrations in QC and JZ (Žilić et al. 2014). For instance, phenolic compounds may react with carbonyl compounds generated during the Maillard reaction to form adducts, which are subsequently converted into more complex polymers or pigments (Teng et al. 2018). In the ginger‐paozhi (JZ) procedure, gingerol compounds, which are abundant in ginger juice, exhibit potent antioxidant properties. These compounds may inhibit the stability of the original phenolic metabolites in CG or transform them into other products by scavenging free radicals or reacting with carbonyl intermediates. This mechanism explains the more pronounced decrease in their content observed in JZ samples (Yang 2018). Alkaloids are more stable than Flavonoids; however, at high temperatures, some Alkaloids may undergo hydrolysis, reducing their content. Additionally, components in ginger juice may affect Alkaloids' stability through enzymatic actions or by altering the matrix's pH, leading to a relatively lower Alkaloid content in JZ (Cho et al. 1988). Benzene and substituted derivatives are volatile compounds that are prone to thermal degradation. During the stir‐frying and ginger‐paozhi procedures, the elevated temperature may cause them to be lost in large quantities through thermal evaporation. Moreover, the benzene ring structure tends to participate in other reactions at high temperatures to form more stable derivatives. These two factors together result in their relatively low contents in JZ and QC (Martins et al. 2000). Studies have shown that lignans exhibit relatively high stability during heat treatment. Under dry‐heat conditions similar to stir‐frying (QC), the cell wall structure is damaged, and heat can induce the hydrolysis of lignan glycoside compounds, releasing free lignan compounds, which leads to a significant increase in their content (Berenshtein et al. 2024). In summary, it is reasonable to infer that processing is beneficial for increasing the content of major drug‐like metabolites, thereby enhancing the medicinal value of QC and JZ as traditional Chinese medicine decoction pieces.

### 3.4 Multivariate Data Analysis of Durg‐Like Metabolites

**FIGURE 5:**
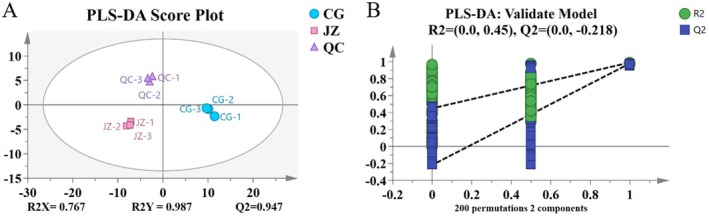
PLS‐DA analysis. (A) Score graph; (B) Validation plot for model replacement test.

To intuitively explore the differences between samples and identify the specific drug‐like components contributing to these variations, PLS‐DA was applied to analyze the drug‐like compounds. The model employed UV scaling for data preprocessing and utilized the default 7‐fold cross‐validation strategy in SIMCA 16 software. The fitting parameters were R^2^X = 0.767, R^2^Y = 0.987, and *Q* ^2^ = 0.947, indicating that the model is reliable. The PLS‐DA score plot of the drug‐like components is shown in Figure 5A. The large distance between sample groups indicates high inter‐group variability and a clear trend in metabolite classification, while the even distribution and close clustering of samples within each group suggest low intra‐group differences. The CV‐ANOVA test (Table 4) (*p* = 0.00037, *p* < 0.01) and permutation test (*n* = 200) demonstrate that the PLS‐DA model does not suffer from overfitting (intercepts: *R* ^2^ = 0.45, *Q* ^2^ = −0.218), as shown in Figure 5B.

**TABLE 4:** CV‐ANOVA.

PLD‐DA	SS	df	MS	F	*p*	SD
Total corr.	16	16	1			1
Regression	15.05	8	1.88	15.93	0.00037	1.37
Residual	0.95	8	0.12			0.34

### 3.5 Screening and Analysis of Different Drug‐Like Metabolites

**FIGURE 6:**
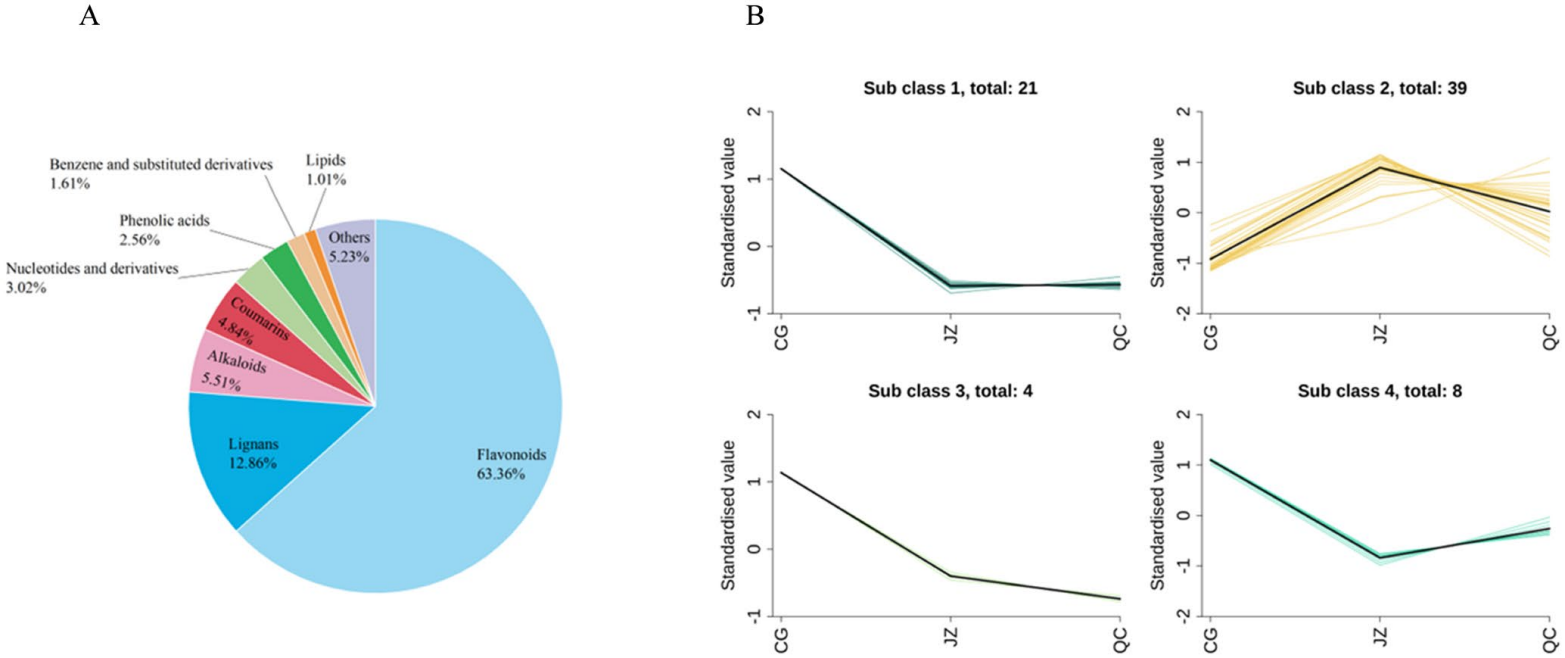
Statistical analysis of the components of different drug properties. (A) Relative content ratio; (B) relative content change trend chart.

To further pinpoint the compounds responsible for the differences in drug‐like properties among CG, JZ, and QC, a screening was performed using the criteria VIP > 1, *p* < 0.05, and log[FC] ≥ 2, resulting in the identification of 72 differential drug‐like metabolites. These included Flavonoids (18 metabolites), Lignans (7 metabolites), Alkaloids (11 metabolites), Coumarins (4 metabolites), Nucleotides and derivatives (3 metabolites), Phenolic acids (5 metabolites), Benzene and substituted derivatives (3 metabolites), Lipids (3 metabolites), Quinones (3 metabolites), Terpenoids (7 metabolites), and Others (8 metabolites). As shown in Figure 6A, Flavonoid compounds comprised the largest proportion (63.36%), followed by Lignans (12.86%) and Alkaloids (5.51%); the remaining classes were present in relatively low amounts, indicating that Flavonoids were most significantly affected by processing. Based on the trends in relative content (Figure 6B), these 72 metabolites were roughly classified into four categories: the metabolites in categories 1, 3, and 4 (a total of 33 metabolites) decreased to varying extents after processing, while those in category 2 (39 metabolites) showed a marked increase. The detailed changes in the relative contents of various types of drug‐like metabolites are shown in Table S3.

#### 3.5.1 Flavonoids

**FIGURE 7:**
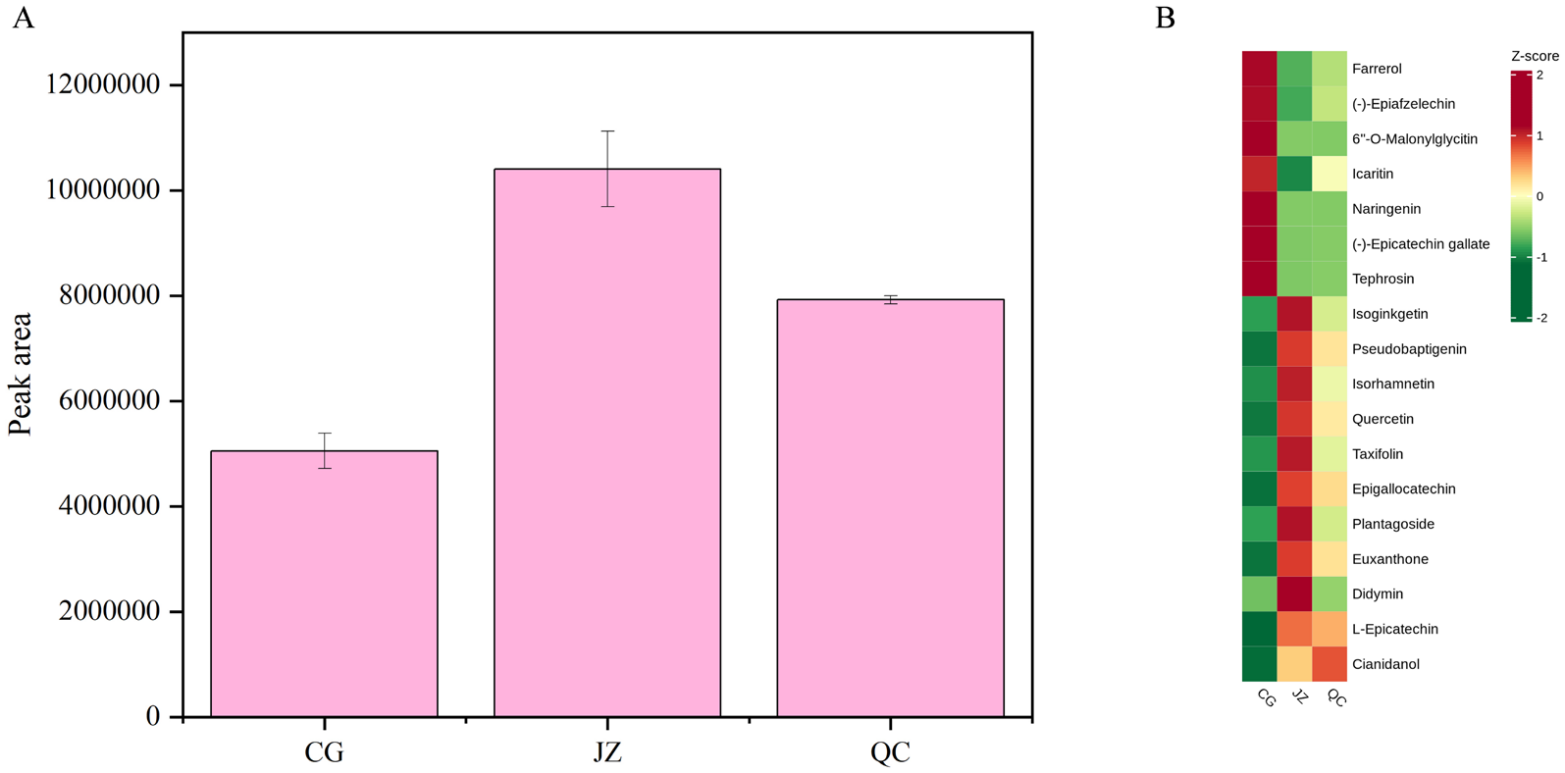
Difference drug‐like flavonoids component statistical analysis. (A) The change in relative content of compounds of differences flavonoids; (B) the change of relative content of all compounds in differences flavonoids.

Flavonoids accounted for 38.17% of the relative content of drug‐like components, making them the main drug‐like components. Meanwhile, they were also the components most affected by processing and played a crucial role in terms of medicinal value. As shown in Figure 7A, which presents the changing trend of Flavonoids' relative content, JZ had the highest relative content, followed by QC, and CG had the lowest. The drying process of CG is similar to a slow thermal degradation process. Although the temperature is relatively low, long‐term heat exposure can cause the cleavage of C‐O bonds and may trigger further skeleton cleavage, resulting in the lowest content of flavonoid compounds in CG (Maflahah et al. 2023). In contrast, QC undergoes high‐temperature stir‐frying for a short period, which rapidly damages the cell wall structure—releasing flavonoid compounds that were originally tightly bound to the cell wall. Additionally, high temperature may induce the hydrolysis of Flavonoid C‐O bonds, converting flavonoid glycosides into flavonols that are easier to extract and detect. As a result, QC exhibits a higher relative content of flavonoid compounds (Toontom et al. 2022). The extremely short heating time of ginger‐paozhi reduces thermal degradation and the maillard reaction. Furthermore, the moistening process may promote the penetration of strong antioxidant components (such as gingerol and curcumin) from ginger juice into the CG tissue, which better inhibits the oxidative degradation of flavonoids (Katsube et al. 2009; Sun et al. 2015).

Flavonoid compounds are known for their warm medicinal properties. The experimental findings that flavonoid compounds levels in JZ and QC are higher than those in CG corroborate the traditional theory that the “warming and dispelling cold” effect of CG is enhanced after processing (Qin et al. 2023). Modern studies have demonstrated that flavonoid compounds possess antioxidant, antidiabetic, and gastrointestinal regulatory effects (Hu, Gao, Su, et al. 2023; Hu, Gao, Zhao, et al. 2023; Huang et al. 2025; He, Wu, et al. 2023), suggesting a potential sequential enhancement of pharmacological activities in CG, QC, and JZ. Furthermore, the variation in flavonoid compounds content between JZ and QC indicates that flavonoid compounds, as one of the main components of ginger, have been transformed and incorporated into the TCM decoction pieces (Engelhardt 2012; Lin et al. 2024; Liu 2024). Short‐term high‐temperature stirring promotes the relative stability and solubility of certain drug‐like flavonoid compounds, while prolonged exposure to high temperatures can lead to their decomposition (Xu et al. 2024).

The specific changes in the relative contents of flavonoid compounds are illustrated in Figure 7B. The relative contents of Farrerol, (‐)‐Epiafzelechin, 6″‐O‐Malonylglycitin, Icaritin, Naringenin, (‐)‐Epicatechin gallate, and Tephrosin were the highest in CG but decreased significantly after processing. Conversely, the relative contents of Isoginkgetin, Pseudobaptigenin, Isorhamnetin, Quercetin, Taxifolin, Epigallocatechin, Plantagoside, Euxanthone, Didymin, and L‐Epicatechin increased significantly after processing, with the highest relative content observed in JZ. This indicates that these compounds are greatly affected by processing with auxiliary materials (such as ginger juice). The relative content of cianidanol also increased after processing but reached the highest level in QC. This suggests that while this metabolite is significantly affected by processing, the impact of auxiliary materials on it is minimal.

In summary, flavonoid compounds are significantly affected by processing, and the relative contents of major flavonoid compounds increase markedly after processing.

#### 3.5.2 Lignans

Lignan compounds are a group of phenolic compounds widely found in nuts, vegetables, and fruits, especially abundant in seeds, possessing antioxidant, antibacterial, and antitumor activities (Berenshtein et al. 2024; Runeberg et al. 2023). In this study, lignans accounted for only 8.17% of the total drug‐like components, but they were the second most affected by processing, indicating their significant impact on the drug‐like properties of CG, QC, and JZ.

**FIGURE 8:**
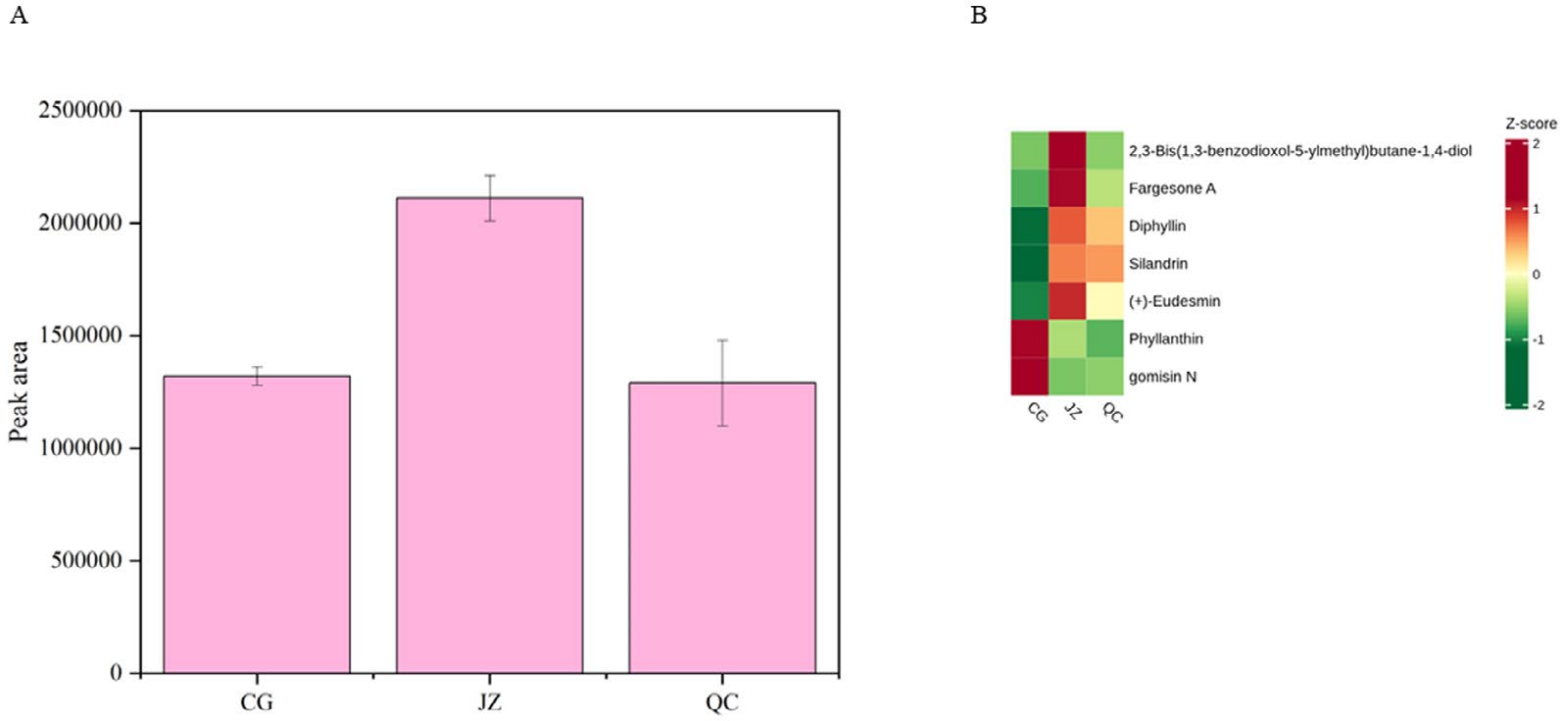
Difference drug‐like lignans component statistical analysis. (A) The change in relative content of compounds of different lignans; (B) the change of relative content of all compounds in different lignans.

The variation trend of lignan compounds is shown in Figure 8A: the relative content is highest in JZ, slightly higher than QC in CG. This may be related to the fact that hydroxyl, carbonyl, and methyl groups on the benzene ring of lignans are easily removed during high‐temperature processing, damaging their structure (Wu et al. 2023). Additionally, reactions related to lignans mainly occur around 120°C, suggesting that lignan components in ginger can be better transformed into JZ at this temperature (Runeberg et al. 2023).

The specific lignan compounds compound variation is shown in Figure 8B: 2,3‐Bis(1,3‐benzodioxol‐5‐ylmethyl)butane‐1,4‐diol, Fargesone A, Diphyllin, Silandrin, and (+)‐Eudesmin have the highest relative content in JZ, followed by QC, and lowest in CG, indicating that these compounds are less affected by processing without excipients and more affected by processing with excipients, with a significant increase after the latter. In contrast, Phyllanthin and Gomisin N are most abundant in CG and lower in JZ and QC, indicating that processing reduces the relative content of these two metabolites. In summary, the relative content of lignan compounds is greatly affected by the processing method with excipients for a short time (3 min) and lower temperature (140°C), and the relative content of most lignan compounds will increase significantly.

### 3.6 Construction of Evaluation Model for Drug‐Likeness Base on PCA

#### 3.6.1 PCA

**FIGURE 9:**
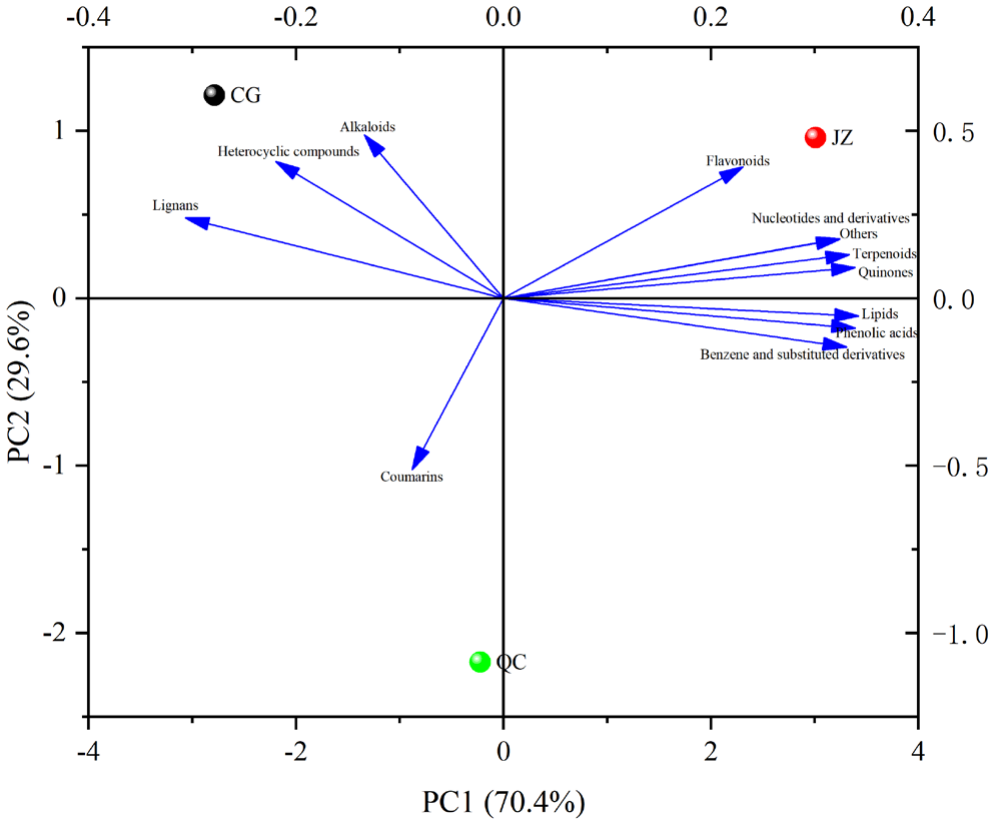
Composite plot of principal component analysis load‐score.

The relative contents of the 12 categories of drug‐like components (classified from 108 drug‐like components) were taken as variables for data standardization. Subsequently, PCA was performed on the standardized data using Origin 2024, and the results are presented in Tables 5, 6, and Figure 9. As shown in Table 5, the eigenvalues of the two principal components were 8.44 and 3.56, with variance contribution rates of 70.35% and 29.65%, respectively. The cumulative variance contribution rate reached 100%, indicating that these two principal components can effectively explain the original data.

**TABLE 5:** Characteristic value and contribution rate of principal component.

Principal component number	Eigenvalue	Percentage of variance (%)	Cumulative (%)
1	8.44	70.35	70.35
2	3.56	29.65	29.65

**TABLE 6:** Load factors for two principal components.

Compounds	PC1	PC2
Alkaloids	−0.13	0.49
Benzene and substituted derivatives	0.33	−0.15
Coumarins	−0.09	−0.51
Flavonoids	0.23	0.39
Heterocyclic compounds	−0.22	0.41
Lignans	−0.31	0.24
Lipids	0.34	−0.05
Nucleotides and derivatives	0.32	0.18
Phenolic acids	0.34	−0.09
Quinones	0.34	0.09
Terpenoids	0.33	0.13
Others	0.32	0.18

Loading coefficients reflect the influence of various drug‐like substances on principal components, with values ranging between −1 and 1. A larger absolute value indicates a greater contribution of the indicator to the principal component (Wu, Zhang, Yan, et al. 2022). According to the data presented in Table 6, PC1 exhibits positive correlations with Lipids (0.34), Phenolic acids (0.34), and Quinones (0.34). It also shows positive correlations with Benzene and substituted derivatives (0.33), Terpenoids (0.33), as well as Nucleotides and derivatives (0.32) and Others (0.32). This suggests that PC1 reflects these indicators with a certain degree of similarity. In contrast, PC2 has positive correlations with Alkaloids (0.49), Heterocyclic compounds (0.41), and Flavonoids (0.39), while showing a negative correlation with Coumarins (−0.51). These results indicate that PC2 can effectively reflect the characteristics of the aforementioned four indicators.

In the PCA loading plot, the closer the variables are to each other, the stronger their positive correlation. As shown in Figure 9, Nucleotides and derivatives, Others, Terpenoids, and Quinones exhibited strong intercorrelations; similarly, Lipids, Phenolic acids, and Benzene and substituted derivatives also showed strong mutual correlations. In the PCA score plot, a greater distance between samples indicates higher variability and a more pronounced trend in metabolite classification. Figure 9 demonstrates that CG, QC, and JZ are well separated, consistent with the findings in Section 3.4. These results indicate that PCA based on the 12 categories of drug‐like components can effectively distinguish between CG, QC, and JZ.

**FIGURE 10:**
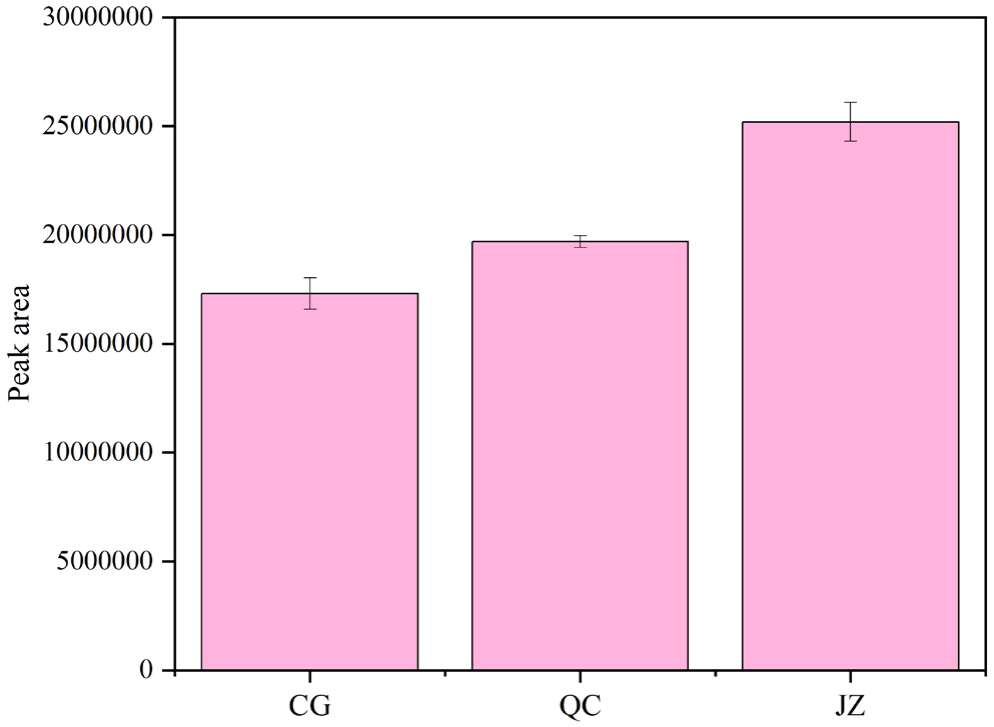
The relative contents of the drug‐like metabolites of CG, QC, and JZ.

Based on Tables 5 and 6, and considering the information represented by the 12 categories of drug‐like component indicators in PC1 and PC2, two new indicators (PC1score and PC2score) were constructed. Subsequently, using the cumulative variance contribution rates of PC1 and PC2 as weights, the comprehensive scores of CG, JZ, and QC were calculated through a weighted method (Table 7). The results showed that CG had a comprehensive score of −1.6, ranking 3rd; JZ had a comprehensive score of 0.89, ranking 1st; and QC had a comprehensive score of 1.60, ranking 2nd. Combined with the analysis of Figure 10, it was found that the higher the comprehensive score (i.e., the higher the comprehensive ranking) of a sample, the higher the relative content of its drug‐like substances. Therefore, it is reasonably inferred that a higher comprehensive score indicates a richer accumulation of drug‐like substances in the sample and better drug‐like potential. Consequently, JZ exhibits the strongest drug‐like, followed by QC, while CG shows the weakest drug‐like.

**TABLE 7:** Comprehensive score of drug‐like properties.

Group	PC1 score	PC2 score	Comprehensive score	Sort
CG	−2.79	1.21	−1.60	3
JZ	3.01	0.96	0.89	1
QC	−0.22	−2.17	0.00	2

Ginger juice is rich in potent natural antioxidants such as gingerol and curcumin. These antioxidants can effectively scavenge free radicals generated during ginger processing, inhibit oxidative degradation, and thereby preserve more active components. Additionally, the active components in ginger may exert a synergistic effect with flavonoids, volatile oils, and other substances in CG, further enhancing the overall anti‐inflammatory and antioxidant capacities (Mao et al. 2019). Short‐term high‐temperature conditions may promote the degradation of some flavonoids. However, due to the extremely short exposure time, oxidative enzymes are rapidly inactivated, preventing further enzymatic oxidation. Conversely, high temperature can break down cell walls, releasing flavonoid glycosides or flavonols that were originally difficult to extract and further promoting the hydrolysis of flavonoid glycosides into flavonols with higher biological activity (El‐Refai et al. 2017). Drying treatment may cause damage to the cell structure of *A. tsaoko*, which activates oxidative enzymes and promotes their massive secretion—this leads to the enzymatic oxidation and decomposition of flavonoid components into colorless products. Additionally, although the temperature of drying treatment is not high, long‐term heat exposure can cause the cleavage of phenolic hydroxyl groups in flavonoid molecules or trigger the Maillard reaction with sugars. This reaction generates brown pigments while consuming active components (Beristain‐Bauza et al. 2019).

### 3.7 Identification of Key Aroma Metabolites Based on ROAV

To identify the key volatile aroma compounds in CG, QC, and JZ, the ROAV method was applied to quantify the contribution of different volatile substances to the overall flavor of the samples and evaluate the role of each volatile component in the total flavor, based on GC–MS data. According to the formula calculation, a total of 44 aroma substances with ROAV ≥ 0.1 were identified (Table 8). Specifically, JZ, CG, and QC contained 37, 18, and 15 important aroma substances (0.1 ≤ ROAV < 1), respectively, as well as 8, 9, and 15 key aroma substances (ROAV ≥ 1), respectively. These aroma substances mainly belonged to eight categories, including Alcohols (4 metabolites), Phenols (1 metabolite), Ethers (1 metabolite), Aldehydes (11 metabolites), Terpenoids (9 metabolites), Ketones (8 metabolites), Heterocyclic compounds (5 metabolites), and Esters (5 metabolites).

**TABLE 8:** ROAV ≥ 0.1 of aroma metabolites.

Number	Compounds	Class	Threshold (mg/g)	ROAV			Aroma characteristics
				CG	JZ	QC	
1	2‐Furfurylthiol	Alcohols	0.006	0.56	—	0.48	Sulfury, roasted, coffee
2	2‐Furanmethanethiol, 5‐methyl‐	Alcohols	0.05	0.10	0.04	0.10	Sulfury, roasted, coffee
3	4‐Phenyl‐2‐butanol	Alcohols	4.3	< 0.01	0.15	0.01	Floral, peony, foliage
4	Benzenemethanethiol	Alcohols	0.0035	1.58	—	1.57	Sharp, alliaceous, onion
5	Phenol, 3‐ethyl‐	Phenols	0.85	0.03	0.65	0.03	Musty
6	Anethole	Ethers	15	0.06	1.93	0.08	Sweet, exotic, flowery
7	2,6‐Nonadienal, (E,Z)‐	Aldehydes	0.01	0.03	1.20	0.05	Cucumber, green
8	2‐Nonenal	Aldehydes	0.1	0.01	0.28	0.02	Fatty, green, waxy
9	2‐Dodecenal, (E)‐	Aldehydes	7.3	0.01	0.40	0.01	Citrus, metallic, mandarin
10	2,4‐Decadienal, (E,Z)‐	Aldehydes	0.07	< 0.01	0.13	< 0.01	Fried, fatty, geranium
11	2,4‐Undecadienal	Aldehydes	0.01	0.04	2.23	0.07	Green, buttery, spicy
12	2‐octenal	Aldehydes	0.2	1.13	7.70	1.49	Fatty, green, herbal
13	(Z,Z)‐3,6‐Nonadienal	Aldehydes	0.05	0.02	0.26	0.02	Fatty, soapy, cucumber
14	Decanal	Aldehydes	0.1	0.03	0.69	0.04	Soapy, waxy, aldehydic, citrus, green
15	2‐Nonenal, (E)‐	Aldehydes	0.08	0.01	0.35	0.02	Fatty, green, cucumber
16	2‐Decenal, (E)‐	Aldehydes	5	< 0.01	0.10	< 0.01	Waxy, fatty, earthy
17	2‐Octenal, (E)‐	Aldehydes	3	0.08	0.51	0.10	Fresh, cucumber, fatty
18	Germacrene D	Terpenoids	1.2	0.03	0.57	0.02	Woody, spice
19	2‐Cyclohexen‐1‐ol, 2‐methyl‐5‐(1‐methylethenyl)‐, acetate	Terpenoids	1.5	0.01	0.31	0.01	Green, minty, spearmint
20	Citronellol	Terpenoids	40	0.01	0.24	0.01	Floral, rose, lime
21	Citral	Terpenoids	100	0.01	0.48	0.02	Sharp, lemon, sweet
22	2,6‐Octadienal, 3,7‐dimethyl‐, (E)‐	Terpenoids	28	0.01	0.34	0.01	Citrus, lemon
23	(1R)‐2, 6, 6‐Trimethylbicyclo[3.1.1]hept‐2‐ene	Terpenoids	5.3	0.21	0.04	0.26	Harsh, terpene, aromatic
24	Eucalyptol	Terpenoids	15	0.12	0.50	0.12	Eucalyptus, herbal, camphor
25	2‐Methylisoborneol	Terpenoids	0.48	0.10	2.50	0.10	Earthy, musty
26	Geraniol	Terpenoids	6.6	0.01	0.29	0.01	Sweet, floral, fruity
27	5,9‐Undecadien‐2‐one, 6,10‐dimethyl‐, (E)‐	Ketones	10	0.01	0.37	0.01	Fresh, green, fruity
28	3‐Octen‐2‐one	Ketones	0.03	14.80	—	15.62	Earthy, spicy, herbal
29	Cyclohexanone, 2,2,6‐trimethyl‐	Ketones	0.1	2.84	12.36	2.90	Pungent, thujone, labdanum
30	1‐Nonen‐3‐one	Ketones	0.001	1.16	21.54	—	Pungent, mushroom
31	Ethanone, 1‐(2‐thienyl)‐	Ketones	1	0.01	0.13	0.01	Sulfury, nutty, hazelnut
32	(5Z)‐Octa‐1,5‐dien‐3‐one	Ketones	0.003	100.00	23.99	100.00	Geranium
33	2 (5H)‐Furanone, 5‐ethyl‐3‐hydroxy‐4‐methyl‐	Ketones	0.002	1.26	31.76	1.22	Sweet, fruity, caramel
34	1,2‐Cyclopentanedione, 3‐methyl‐	Ketones	26	0.03	0.16	0.03	Burnt, caramel, fragrant
35	Pyrazine, 2‐methoxy‐3‐(1‐methylethyl)‐	Heterocyclic compounds	0.002	0.45	—	0.47	Beany, pea, earthy
36	Pyrazine, 2‐methoxy‐3‐(1‐methylpropyl)‐	Heterocyclic compounds	0.002	0.12	2.18	0.08	Musty, green, pea, galbanum
37	Pyrazine, 2‐methoxy‐3‐(2‐methylpropyl)‐	Heterocyclic compounds	0.002	2.85	100.00	6.18	Green bell pepper, pea
38	Thiazole, 2,4‐diethyl‐	Heterocyclic compounds	6.5	0.04	0.16	0.04	Earthy, ethereal, musty
39	2‐Methoxy‐3,5‐dimethylpyrazine	Heterocyclic compounds	0.0004	46.91	14.26	50.03	Bread, mousy
40	Butanoic acid, 3‐methyl‐, 2‐phenylethyl ester	Esters	0.01	0.05	1.93	0.04	Floral, fruity, sweet
41	3‐Mercaptohexyl acetate	Esters	0.02	0.04	1.64	0.05	Sulfury, grapefruit, fruity
42	Pentanoic acid, 2‐methyl‐, ethyl ester	Esters	0.003	0.86	—	1.06	Fresh fruit, green, melon
43	Benzene, (isothiocyanatomethyl)‐	Esters	0.7	0.12	4.35	0.16	Mild, watercress, dusty
44	cis‐3‐Hexenyl isovalerate	Esters	20	< 0.01	0.14	< 0.01	Fresh, green, apple

**FIGURE 11:**
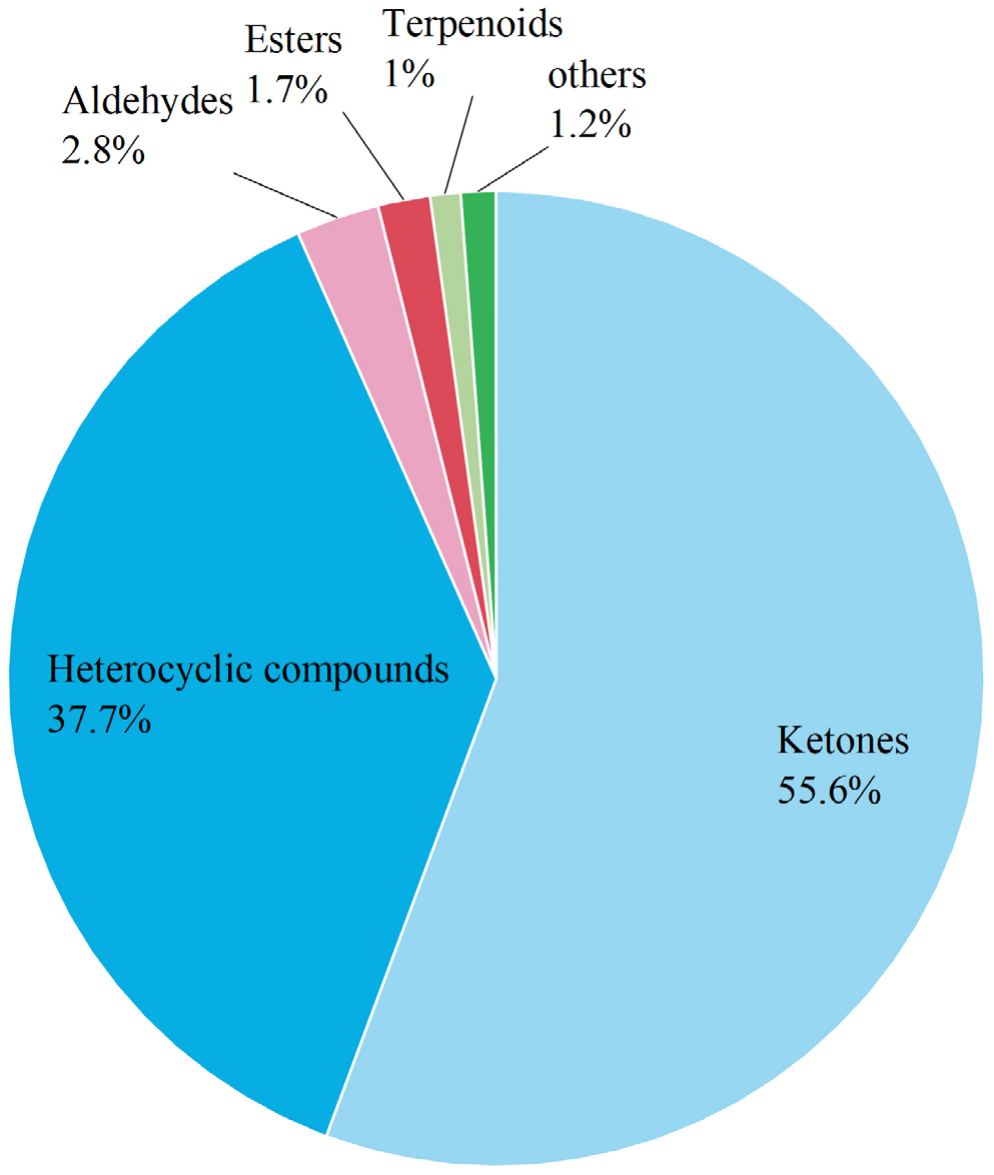
Proportion of ROAV value of 44 aroma metabolites.

The relative contribution ratios of ROAV values for various aroma substances are shown in Figure 11. Ketones accounted for the largest proportion of ROAV values at 55.6%, followed by heterocyclic compounds at 37.30%, while the proportions of the remaining categories were relatively low. In numerous studies on spices, plant essential oils, and volatile compounds, ketones are often recognized as one of the compound categories with the greatest aroma contribution, and heterocyclic compounds also make significant contributions to aroma (Ma et al. 2024; Tu et al. 2022).

### 3.8 Analysis of ROAV and QDA

**FIGURE 12:**
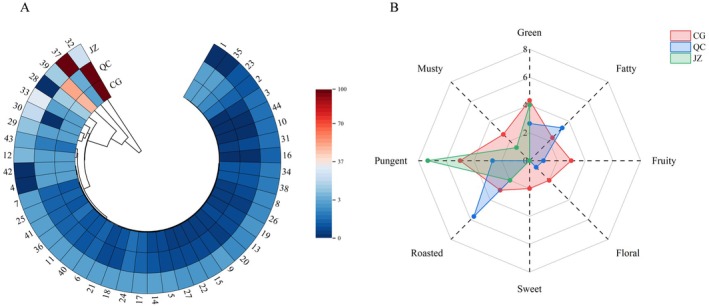
Analysis of aroma characteristics. (A) Heat map of ROAV value changes for 44 aroma substances; (B) aroma attribute radar chart. The numbers in Figure 12A represent the compounds in Table 8.

The ROAV heatmap of the 44 aroma substances (Figure 12A) intuitively reflects the contribution degree of different aroma substances to the samples—the more reddish the color, the greater the contribution. Specifically, aroma substance No. 32 ((5Z)‐Octa‐1,5‐dien‐3‐one) exhibited a ROAVstan = 100 in the CG and QC, indicating the largest contribution to the overall flavor, primarily presenting a geranium‐like aroma. In contrast, aroma substance No. 37 (Pyrazine, 2‐methoxy‐3‐(2‐methylpropyl)‐) showed a ROAVstan = 100 in the JZ, contributing the most to the overall flavor, primarily presenting green bell pepper and pea‐like aromas. Based on eight aroma attributes, with aroma intensity as the evaluation criterion, a quantitative descriptive sensory evaluation of aroma was conducted for the CG, QC, and JZ samples. The aroma characteristic radar chart (Figure 12B) shows that the flavor profiles of the three samples differed significantly: CG exhibited a relatively balanced aroma profile, dominated by a pungent note and supplemented by a green note; QC was characterized by a roasted note, accompanied by fatty, green, and pungent notes; JZ was dominated by a pungent note, with a green note as a supplement.

ROAV analysis showed that green‐type compounds exhibited the highest total ROAV among the three samples, indicating that these substances are the key basic components forming the aroma characteristics. However, the results of quantitative descriptive sensory analysis revealed that the sensory intensities of roasted and pungent notes were significantly higher than that of the green note. Studies have shown that due to its mild odor properties and weak penetration, the green note does not dominate the overall aroma profile (Zhao et al. 2023). Moreover, during processing and heating, the green note is easily masked by stronger, more recognizable aroma notes such as roasted and pungent. Therefore, the roasted and pungent notes of the samples are more dominant in sensory analysis (Liang et al. 2024; Wu et al. 2024; Zhang et al. 2023).

Drying treatment primarily reduces water activity by evaporating moisture. This mild heat treatment method retains the original volatile oil components in *A. tsaoko*, and moreover, due to the relatively low temperature, no significant Maillard reaction is triggered. As a result, the roasted or fatty aromas are relatively weak, and the sample mainly exhibits the original pungent note and fresh green note (Qin et al. 2022; Yang et al. 2008). The pyrazine and furfural compounds generated by the intense Maillard reaction during the stir‐frying process are the main sources of the roasted note. Meanwhile, fatty acid ester compounds produced by lipid oxidation under high temperature impart a fatty note to the QC sample (De Vleeschouwer et al. 2010; Qin et al. 2021; Zhou et al. 2016). The addition of ginger juice inhibits the progression of lipid oxidation and the Maillard reaction to a certain extent, thereby reducing the formation of roasted and fatty notes (Hong et al. 2015). Furthermore, the pungent taste of ginger juice itself may interact with 1,8‐cineole in CG, which further enhances the overall pungent note (Starkenmann et al. 2007).

## 4 Conclusion

A total of 1625 and 1145 metabolites were detected through GC–MS and LC–MS, respectively. Among these, the components identified by GC–MS were dominated by terpenoids, while those identified by LC–MS were primarily flavonoids. Based on the components from GC–MS and LC–MS, 108 druggable metabolites were discovered, with flavonoids being the primary druggable components—their relative content increased sequentially in CG, QC, and JZ. There were significant differences in drug‐like properties among CG, QC, and JZ, and a total of 72 differential drug‐like components were screened out. These components could be further divided into four categories according to their relative content variation trends: the relative content of compounds in the second category increased sequentially in CG, QC, and JZ, while the relative content of compounds in the other three categories decreased sequentially in CG, QC, and JZ to varying degrees. The differential druggable components were mainly composed of flavonoids and lignans. For flavonoids, the relative content of most compounds increased in both QC and JZ, with the highest content observed in JZ. For lignans, the relative content of most compounds was the highest in JZ, while it was relatively low in both QC and CG, and there was no significant difference in content between QC and CG. Using 12 categories of metabolites as indicators, a comprehensive evaluation of the drug‐like properties of CG, QC, and JZ was conducted via PCA weight analysis. The results indicated that the drug‐like properties decreased sequentially in JZ, QC, and CG.

Based on the 1165 GC–MS components identified, a total of 44 aroma substances were screened using the ROAV method. By integrating ROAV values with quantitative descriptive sensory evaluation, the aromatic profiles of the samples were comprehensively characterized. For the CG sample, the dominant aroma was characterized as pungent, complemented by a green note, with the key aroma compounds contributing to this profile including 3‐Octen‐2‐one, 2,2,6‐trimethyl‐Cyclohexanone, and (5Z)‐Octa‐1,5‐dien‐3‐one. In contrast, the QC sample was primarily characterized by a roasted aroma, with additional contributions from fatty, green, and pungent notes. The pivotal aroma compounds identified in this sample were 2‐Methoxy‐3,5‐dimethylpyrazine, (5Z)‐Octa‐1,5‐dien‐3‐one, and 3‐Octen‐2‐one. Lastly, the JZ sample exhibited a predominant pungent aroma, supplemented by a green note, with its key aroma compounds comprising 1‐Nonen‐3‐one, 2,2,6‐trimethyl‐Cyclohexanone, and 2‐Methoxy‐3‐(2‐methylpropyl)‐pyrazine.

From the perspective of drug‐like and aroma characteristics, this study systematically linked the paozhi methods to the changes in drug‐like and aroma components across the three sample types. The research clarified the specific drug‐like and aromatic profiles of each sample, thereby providing a solid data foundation for the in‐depth exploration of their medicinal and culinary properties. The ultimate goal is to achieve “symptom‐oriented paozhi” for medicinal applications and “flavor‐oriented paozhi” for culinary uses, while also offering valuable data support for the utilization and development of medicinal and edible resources.

## Author Contributions

**Jinyu Zhang:** funding acquisition, methodology, project administration, resources, data curation, supervision, conceptualization. **Han Chen:** writing – original draft, writing – review and editing, data curation, software, investigation, validation, visualization. **Weize Yang:** data curation, supervision, resources, software. **Meiquan Yang:** visualization, validation, project administration, formal analysis. **Tianmei Yang:** methodology, project administration, resources, supervision, data curation, funding acquisition. **Zongliang Xu:** supervision, data curation, resources. **Mingju Qi:** software, investigation, conceptualization.

## Conflicts of Interest

The authors declare no conflicts of interest.

## Supporting information


**Table S1:** The characteristics of the eight types of aroma attributes.

**Table S2:** The LC–MS components of all samples.

**Table S3:** The durg‐like metabolites of all samples and the categories to which these components belong.

## Data Availability

Date available in article Supporting Information.
